# Cold atmospheric plasma as a promising approach for gelatin immobilization on poly(ε-caprolactone) electrospun scaffolds

**DOI:** 10.1007/s40204-019-0111-z

**Published:** 2019-03-27

**Authors:** Marziyeh Meghdadi, Seyed-Mohammad Atyabi, Mohamad Pezeshki-Modaress, Shiva Irani, Zahra Noormohammadi, Mojgan Zandi

**Affiliations:** 1 Department of Biology, School of Basic Sciences, Sciences and Research Branch, Islamic Azad University, Tehran, Iran; 2 Department of Pilot Nanobiotechnology, Pasteur Institute of Iran, Tehran, Iran; 3 Burn Research Center, Iran University of Medical Sciences, Tehran, Iran; 4 Department of Biomaterial, Iran Polymer and Petrochemical Institute, Tehran, Iran

**Keywords:** Cold atmospheric plasma, PCL, Nanofibers, Grafting, Gelatin

## Abstract

Poly(Ɛ-caprolactone) (PCL) is a biocompatible polymer with a high potential to be used in tissue engineering especially in tight tissues. In the current study, cold atmospheric plasma (CAP) is used as a promising method for immobilization of gelatin as a functional biomacromolecule on PCL nanofibrous substrates. The CAP surface modification leads to oxidation of chemical groups existing on the PCL surface without doing any damage to the bulk properties of biomaterials for gelatin biomacromolecule grafting. The water contact angle (WCA) of the CAP-treated surface and gelatin-grafted PCL using CAP indicates an effective increment in the hydrophilicity of the PCL surface. Also to achieve the highest levels of gelatin grafting on the PCL surface, two different grafting methods and gelatin concentration diversity are utilized in the grafting process. The immobilization of gelatin biomacromolecules onto the CAP surface-modified PCL nanofibers is investigated using scanning electron microscope (SEM) and Fourier transform infrared spectroscopy (FTIR). The gelatin-modified PCL substrates revealed uniform nanofibrous morphology with increased average fiber diameter. The results of FTIR spectra, including hydroxyl groups, NH groups, and amide II of gelatin-grafting peaks, confirm the gelatin immobilization on the surface of nanofibers. The metabolic activity of cultured mesenchymal stem cells (MSCs) on the surface-modified scaffolds is evaluated using MTT analysis (*P ≤ *0.05). The results of metabolic activity and also SEM and DAPI staining observations indicate proper attachment on the surface and viability for MSCs on the surface-immobilized nanofibrous scaffolds. Therefore, CAP treatment would be an effective method for biomacromolecule immobilization on nanofibers towards the enhancement of cell behavior.

## Introduction

Tissue engineering is a promising approach for regenerating injured tissues. Within the field, scaffolds, as crucial components, play a fundamental role (Lannutti et al. 2007). An ideal scaffold acts like an extracellular matrix (ECM) to provide a three-dimensional (3-D) structure with a controlled degradable rate for cell adhesion and proliferation as well as expressing a specific phenotype (Courtney et al. 2006; Qi et al. 2016). Thus, selecting an appropriate scaffold is of the critical steps towards the successful tissue-engineered structure.

Furthermore, another challenging field is selecting the best technique for scaffold fabrication. Among various scaffold fabrication techniques in the realm of tissue engineering, electrospinning has attracted increased attention. Electrospinning may be utilized to fabricate nanofibrous scaffolds to imitate the natural extracellular matrix and also porous architecture structure to enhance the cellular infiltration (Pezeshki-Modaress et al. 2015a, 2017). The electrospun poly(ε-caprolactone) (PCL) scaffold is one of the most widely used synthetic polymers due to its excellent biodegradability, biocompatibility, and mechanical properties (Dabouian et al. 2018). Although PCL-based electrospun scaffold exhibits desirable characteristics, inherent hydrophobic nature of PCL limits the tissue engineering applications including cell adhesion, growth, and differentiation due to lack of adsorption of cell-adhesive proteins such as fibronectin, vitronectin, and collagen from the biological serum (Beachley and Wen 2010; Cipitria et al. 2011; Pham et al. 2006).

To overcome these limitations, there are some physical and chemical techniques to modify the surface leading to the creation of specific functional groups on the surface of the scaffolds and increasing the hydrophilicity and cell adhesion (Ma et al. 2007). Surface hydrolysis using the NaOH solutions and surface aminolysis through hexanediamine are two applied chemical modification methods. These techniques lead to the introduction of some functional groups on the polyester surfaces to improve the cellular interactions; but they are very time-consuming and may result in some undesirable effects such as loss of mechanical properties and faster degradation process (Oyane et al. 2005; Yu et al. 2009). Physical surface modification methods such as peroxide oxidations, ozone oxidations, gamma rays, and ultraviolet radiation may also introduce reactive functional groups on the scaffold surface. However, degradation of polymers and usually non-permanent effects may appear in physical techniques (Koo and Jang 2008). Plasma is another physical method to modify the polymer surface. Compared to the other described chemical and physical techniques, plasma surface modification is a very suitable technique for creating reactive functional groups on the surface of the biodegradable scaffolds without changing the beneficial bulk properties. Besides introducing the functional groups, plasma-based techniques are solvent-free which can uniformly treat the surface of the scaffolds and can also be employed for immobilization of the protein or other biochemical molecules on the surface of the scaffolds. After all, the high potential of plasma in the modification of biodegradable polymers is evident due to its advantages over other techniques (Martins et al. 2009; Zelzer et al. 2009).

Plasma can be subdivided into thermal and non-thermal (or cold) groups. The thermal plasma cannot be used in surface modification of heat-sensitive materials such as polymers, because it is composed of high-temperature electrons and heavy particles. In contrast, non-thermal plasmas are composed of low-temperature particles and relatively high-temperature electrons. The ions and neutrals remain relatively cold and cause the gas temperature to remain constant at room temperature, so it is called “cold atmospheric plasma” (CAP) (Atyabi et al. 2016). This feature allows the utilization of this type of plasma in heat-sensitive materials such as biodegradable polymers (De Geyter et al. 2008; Shahmirani et al. 2014). As mentioned in many transcripts, CAP is one of the most trending approaches to modify and improve the functional and topographical features of the surface. It can be utilized to enhance the biocompatibility of the new scaffolds due to its capability to build extensively reactive functionality and also with no possible toxicity. These functional groups can be used to immobilize the biologically active molecules to provide ligands for cell integrins (Gümüşderelioğlu and Türkoğlu 2002; Şaşmazel et al. 2009; Surucu et al. 2016). CAP technology for improvement of the scaffolds in tissue engineering is studied by Pieter Cools et al. It is mentioned that atmospheric pressure plasma jets (APPJs) are mainly used to generate plasma discharges when the PCL nanofibers are exposed to a plasma treatment (Cools et al. 2016). Ja-Young Jang et al. have also investigated the biomedical applications of the plasma jet. The ROS (reactive oxygen species) and the RNS (reactive nitrogen species) produced by plasma resulting in biological effects which range from the oxidation of lipids and proteins to the induction of cell signaling. Besides, to achieve the desired results, plasma can be set out by adjusting the distance between the device nozzle and the to-be-treated sample and also by selecting the gas mixture (Laroussi 2015). Bárdos et al. introduced helium as a suitable gas to generate plasma discharges due to its physical properties such as the small diameter of gas molecules and, thus, long mean free path compared to other gasses (Bárdos and Baránková 2010).

By the way, induced properties of plasma-treated surfaces are not permanent, but the grafting process makes a permanent grafting. In the process of grafting on the surface, ECM proteins such as gelatin are used to imitate an environment similar to the natural ECM, thereby facilitates the cell–scaffold interactions for tissue engineering (Morent et al. 2008; Sadeghi et al. 2018; Yuan et al. 2012). Zuewi et al. used air plasma to introduce COOH groups on the surface to the covalent grafting of the gelatin molecules (Ma et al. 2005b). The effects of gelatin-grafted scaffolds on the biological behavior of the fibroblastic cells have also been investigated; the gelatin, through extracellular matrix fibronectin glycoproteins, increases the signaling pathway and cell adhesion ligands to improve the cellular attachment and proliferation (Safaeijavan et al. 2014).

In this study, CAP discharge is generated with a plasma jet by applying helium gas as a new contribution to the grafting process. CAP treatment introduces carboxyl and hydroxyl groups on the PCL nanofiber surface, and enables the immobilization of gelatin as the functional biomacromolecules on the nanofibers’ surface.

## Experimental

### Materials and methods

#### Fabrication of electrospun PCL scaffold

To fabricate PCL nanofibers using electrospinning method (CO881007NYI machine, Asia Nanostructure/Iran), a PCL solution (MW80000) was prepared in formic/acetic acid (9:1 ratio) at a concentration of 12.5% (*w*/*v*). The PCL solution was fed into a needle attached to a syringe pump. A rotating cylindrical drum was used as a collector and placed at a distance of 15 cm from the needle with a flow rate of 1 mL/h and a voltage of 25 kV was applied. The collecting rate was 250 rpm. After fabricating the scaffolds, they are cut in 0.5 × 0.5 cm^2^ to be surface-modified. The thickness of the scaffolds is 40 µm.

#### Surface modification

**Fig. 1:**
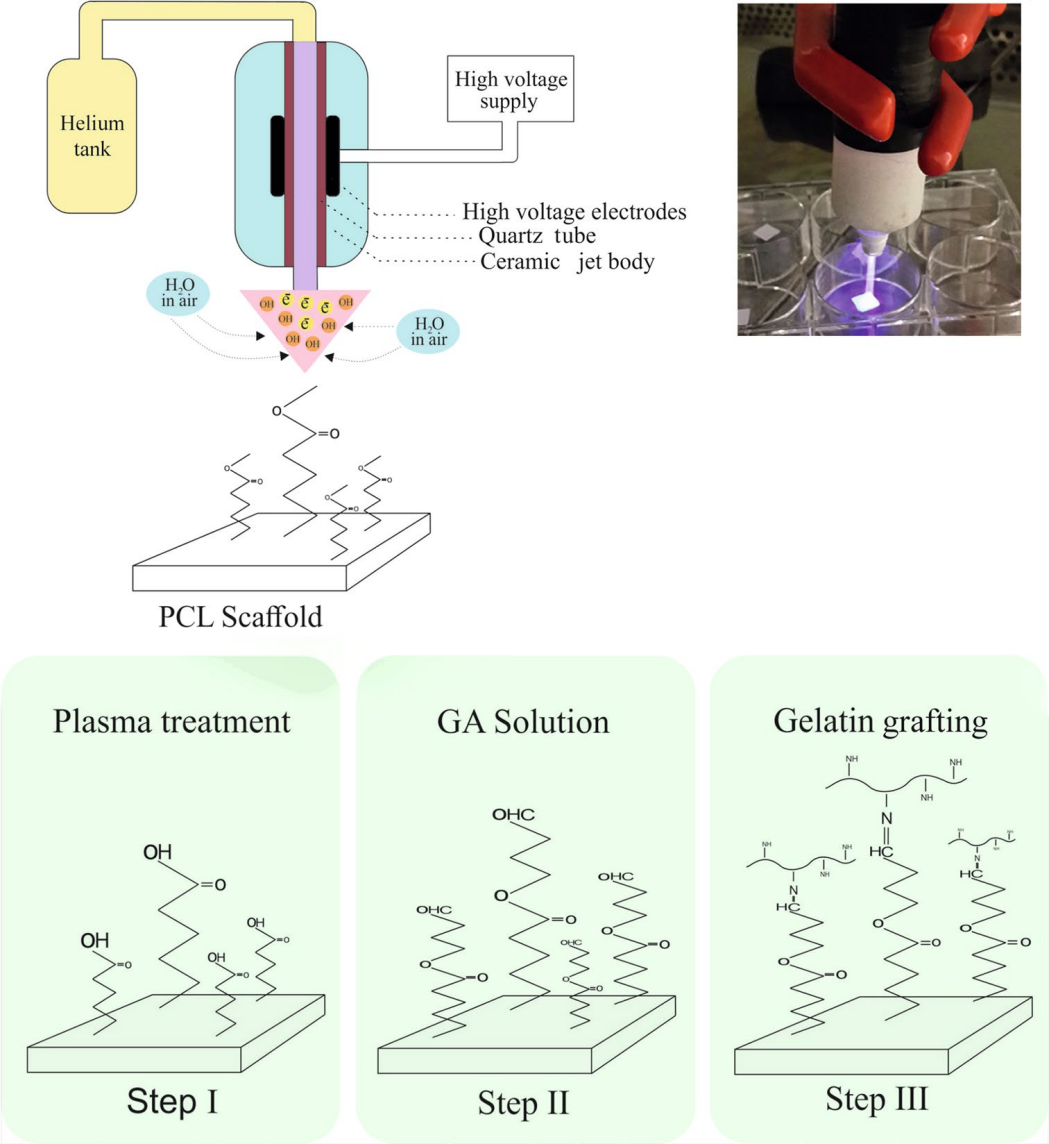
A schematic view of the plasma jet and the reaction of surface-modified PCL nanofibers. CAP treatment is used to introduce carboxyl group on the surface, followed by covalent attachment of gelatin. Glutaraldehyde (GA) is used as linker to covalently immobilized gelatin on CAP-treated PCL scaffold

As shown schematically in Fig. 1, surface modification of PCL nanofibers was performed in two steps: (1) plasma treatment and (2) gelatin grafting.

In the first step, time is chosen to find the best treating duration of PCL scaffold surface; in the second step, concentration is evaluated to understand the best concentration for grafted gelatin on the treated scaffold surface.

#### Step I: CAP treatment and introduction of primary carboxyl onto PCL surface

In this study, The CAP device generated plasma jet into a 4.5 mm-diameter quartz tube as an outer electrode with helium gas flow (flow rate was kept constant at 5 L/min) by applying the high voltage of 13.5 kV at room atmosphere. The helium discharge plasma absorbs the water vapor in the ambient air, and the OH radicals are formed due to the interaction with water vapor. These radicals react with the polymer chains at a distance of 2 cm beneath the plasma electrode. Thus, polar hydrophilic groups such as hydroxyl and carboxyl are formed on the surface of PCL nanofibers (De Geyter et al. 2008). Oxygen-containing groups on the surface of the PCL nanofibers are introduced for improving the grafting of gelatin. Duration of the treatment is one of the most essential factors in the CAP treatment of the scaffold surface. The best duration time for surface treatment of the scaffolds is the time which enables the most functional groups attached on the surface with minimum changing in surface morphology, such that the nanofibers morphology is preserved. For selecting the optimal duration of treatment, scaffolds have been treated for 1, 3, 5, and 7 min.

#### Step II: Immobilization of gelatin onto CAP-treated PCL nanofiber

Gelatin is a natural polymer remarkably similar to collagen, but less sensitive to degradation. Many reports suggest that gelatin derivatives show a great potential to conduct the migration, adhesion, growth, and organization of the cells, and improve cellular interaction with material through cell signaling and adhesion of ligands for cell function. For gelatin grafting on plasma-treated PCL nanofibers, two methods are used. In the first method (method I), plasma-treated PCL nanofibers are immersed in 0.1% glutaraldehyde (GA) solution for 3 h at room temperature. In this method, a covalent bond is formed between oxygen-containing groups generated in the surface of nanofiber and hydroxyl groups in GA solution. The scaffold is then rinsed with distilled water, immersed in different concentrations of gelatin solutions (3.5 and 7 mg/mL in PBS buffer), and kept at 4 °C overnight. Finally, gelatin-grafted PCL nanofiber is rinsed with distilled water for 24 h to eliminate non-covalent immobilized gelatin and dried under vacuum.

In the second method (method II), the plasma-treated nanofibers are first immersed in gelatin solution at different concentrations (3.5 and 7 mg/mL in PBS buffer) and then reacted at 4 °C overnight. The scaffold is rinsed with distilled water, and it is immersed in 0.1% GA solution for 3 h at room temperature. In this method, GA solution is used as a crosslinking agent to stabilize the gelatin biomacromolecules on a CAP-treated surface. Finally, the gelatin-grafted PCL nanofiber is rinsed with distilled water for 24 h and dried under vacuum.

### Surface characterization of modified PCL nanofibers

#### Morphology study

The influence of surface modification on the morphology of electrospun nanofibers is investigated using scanning electron microscopy (SEM, VEGA, TESCAN, Czech) at accelerating voltage of 2 kV after gold sputter coating. The diameter of the fiber is measured from the SEM micrographs by image analysis software.

#### ATR-FTIR spectroscopy

The surface of the scaffolds is investigated by attenuated total reflection-Fourier transform infrared (ATR-FTIR, Perkin Elmer Spectrum RX I, US) to investigate the chemical modification during CAP treatment and gelatin immobilization.

Contact angle measurement

To determine the wettability of the modified PCL nanofibers, the water contact angle is measured by a water droplet on the nanofibers surface after 10 s at room temperature. Pure water of 2 μL is pumped through a syringe on the nanofibers’ surface. Five samples are measured for each test. The average value is reported with standard deviation (SD).

### Biocompatibility evaluations

#### MSCs’ culture and cell seeding on scaffolds

The human mesenchymal stem cells (MSCs) are obtained from the National Cell Bank, Royan Institute of Iran. The MSCs were maintained in DMEM culture medium supplemented with 10% fetal bovine serums (FBS). They were incubated at 37 °C in a humidified atmosphere of 5% CO_2_. A routine subculture was used until the third passage. MSCs of third passage (upon 80% confluence) were harvested by trypsinization by 0.025% trypsin–EDTA and suspended in DMEM culture with 10% FBS.

Before the cell seeding, each side of nanofibrous scaffolds is sterilized by UV radiation for 20 min. Then, MSCs are seeded at a density of 10^4^ cell/well and incubated for 2 h and DMEM culture medium supplemented with 10% FBS is added.

### Assessment of cell morphology and attachment of MSCs on scaffolds

To investigate morphology, attachment, and distribution of the cultured MSCs on modified scaffolds, inverted contrast light microscopy and SEM are used.

For MSCs’ morphology assessment, cultured cells on scaffold were fixed with 2.5% (v/v) glutaraldehyde solution for 2 h, and then dehydrated in series of the sequentially increasing concentration (60, 70, 80, 90, and 100%) of ethanol solutions at 37 °C for 10 min. Finally, the scaffolds were coated with gold and then observed by SEM**.**

### Nuclear staining with DAPI

Cultured MSCs on scaffold were fixed with 4% paraformaldehyde for 30 min, followed by washing with PBS. Next, Triton X-100 (0.3%) was added for 10 min. Finally, after washing with PBS, the nucleus of the cultured cells on the scaffold was labeled with DAPI (4′,6-diamidino-2-phenylindole; 1:1000) and then observed by fluorescent microscope.

### Cell viability

The colorimetric MTT assay was performed to quantify the cell behavior and biocompatibility of the PCL nanofibers using 3-(4,5 dimethyldiazol-2-yl)-2,5-diphenyltetrazolium bromide (MTT, Merck Promega). Briefly, after 24, 48, and 72 h of cell seeding, the old culture medium was replaced with DMEM culture medium-containing 10% MTT dye. The plates were incubated at 37 °C for 4 h leading to a reaction of MTT with mitochondrial dehydrogenases of living cells and forming formazan crystals. Then, MTT supernatant was removed, and DMSO was added to solve formazan crystals. Finally, after shacking in a dark place for 20 min, the absorbance was measured at 570 nm using an enzyme-linked immune sorbent assay (ELISA) plate reader. The experiment was repeated three times, and the results are presented as mean ± SD.

### Statistical analysis

All data in this study are analyzed using ANOVA (SPSS 19.0). *P* values less than 0.05 are considered significant.

## Results

### Characterization of CAP-treated PCL nanofibers

**Fig. 2:**
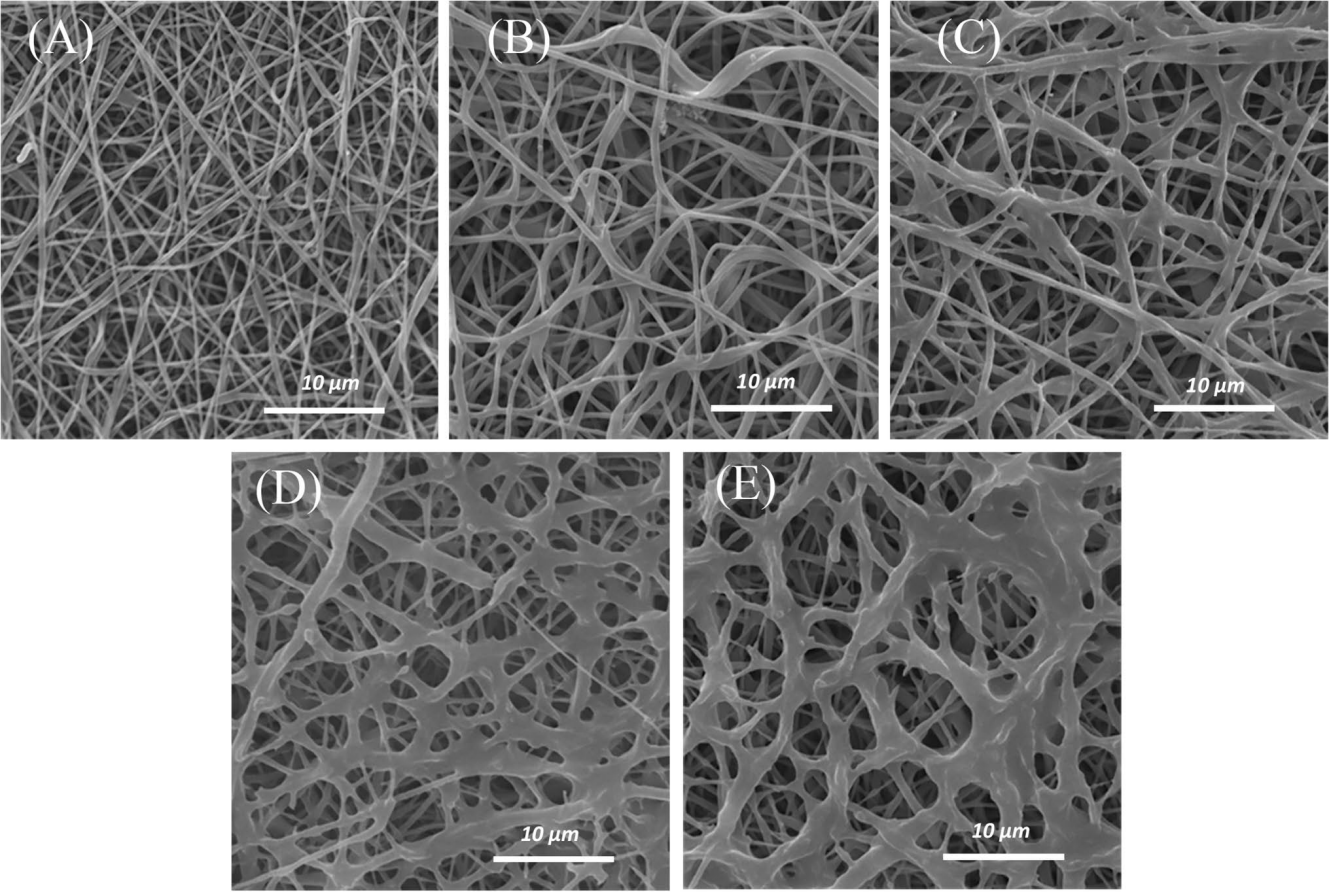
SEM images of unmodified PCL scaffold (**a**), CAP-modified PCL scaffold with 1 min (**b**), 3 min (**c**), 5 min (**d**), and 7 min (**e**) treatment time

**Fig. 3:**
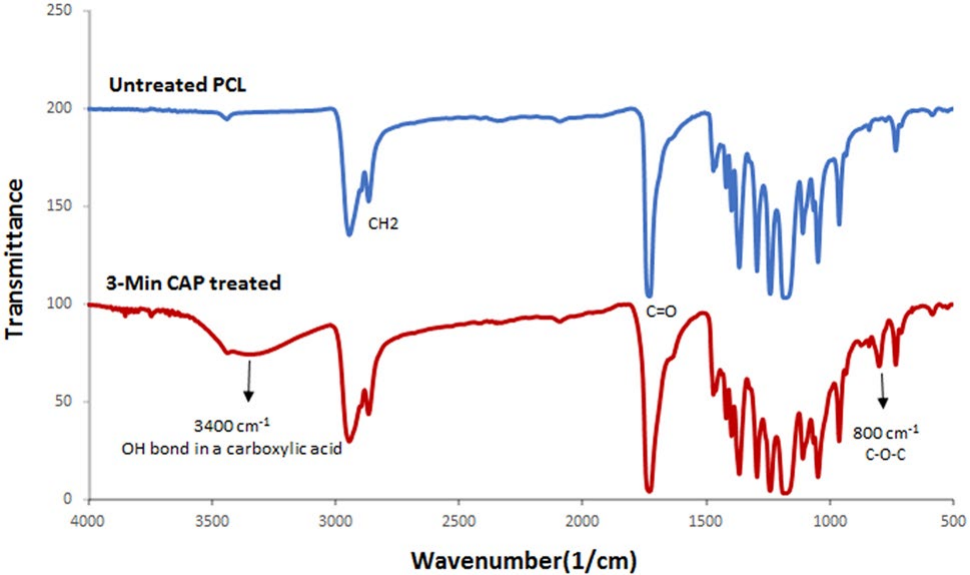
FTIR spectra of unmodified PCL scaffold and CAP-modified PCL scaffold (3 min)

To investigate the morphology of the treated scaffolds, SEM imaging of electrospun PCL nanofibers was provided using electron microscopy (Fig. 2a). According to SEM images, PCL scaffold showed a highly porous fibrous structure with interconnected pores. It can be seen that the fibers were bead-free, with smooth randomly oriented morphology. The average of the fiber diameters was 310 ± 68 nm (Table 1). The influence of CAP-treatment time (1, 3, 5, and 7 min) on nanofibers’ morphology was assessed using SEM micrographs. The SEM results revealed that 1-min (Fig. 2b) and 3-min CAP-treated scaffolds (Fig. 2c) maintained their nanofibrous morphology. However, by exposure to helium plasma for more than 3 min (5 and 7 min), the melted nanofibers of scaffold surface resulted in the aggregation of fibers (Fig. 2d, e). The results (Fig. 2) have revealed that the maximum CAP-treatment time for maintaining the nanofibrous morphology was 3 min. To confirm the effect of CAP treatment (3 min) on the surface of PCL nanofiber, FTIR-ATR technique was used (Fig. 3). The sharp peak of "carbonyl stretching mode" was easily identified at around 1727 cm^−1^ according to this spectrum (Fig. 3). On the other hand, CH_2_ vibrations were the causes of the observed peaks at 2937 and 2866/cm in untreated samples. ATR-FTIR spectra (Fig. 3) have shown the difference between the untreated and CAP-treated samples. After treatment, abroad absorption peak has appeared at 3650 and 3300/cm due to OH-stretching vibrations. The 800/cm peak (out-of-plane deformation) is attributed to interlayer carbonate anions vibrations. The peaks are related to O–C–O and C–O–C lattice vibrations appeared in the range of 500–800/cm (Dolci et al. 2014; Pezeshki-Modaress et al. 2015b).

**Table 1:** Average diameter of PCL and modified PCL nanofibers; average ± SD (n = 100)

Sample	Average diameter (nm)
PCL nanofibers	310 ± 68
Gelatin-grafted PCL nanofibers with a concentration of 5 mg/mL via method I of grafting method	500 ± 170
Gelatin-grafted PCL nanofibers with a concentration of 5 mg/mL via method II of grafting method	400 ± 140

### Gelatin grafting on the CAP-treated scaffolds

**Fig. 4:**
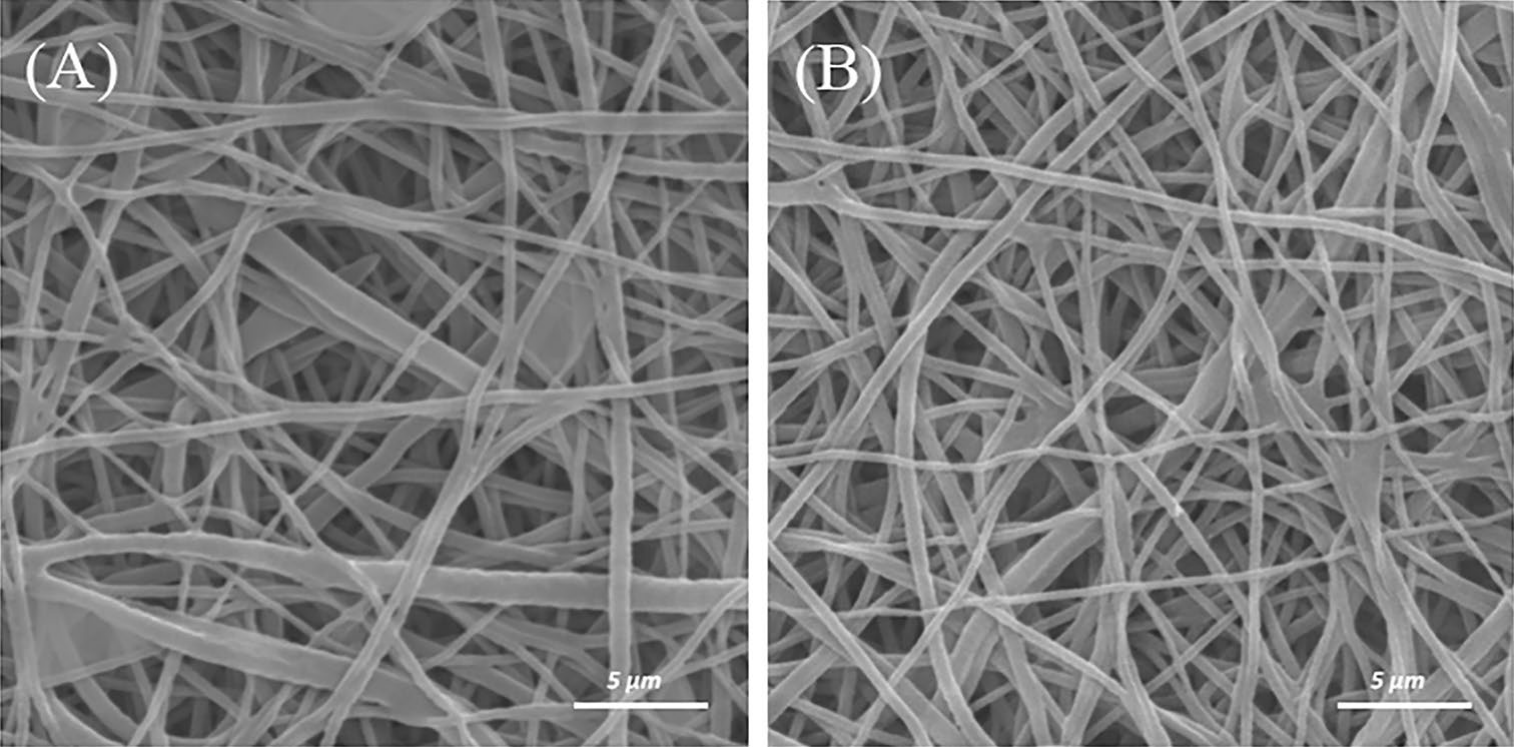
SEM images of gelatin-grafted nanofibers PCL scaffold with a concentration of 5mg/Ml through the method I (**a**) and method II (**b**) on the CAP-treated scaffold (3 min)

CAP-treated scaffolds (3 min) were grafted through two methods (I and II) at constant concentrations of gelatin. To study the influence of how gelatin molecules are grafting (methods I and II) on PCL nanofibers, morphology and average fiber diameter are observed using SEM micrographs (Fig. 4a, b). The SEM images show that the surface of the gelatin-grafted fibers becomes rough and thick due to the grafting layer. Some of the attached gelatin can also be observed between the junctions of the nanofibers especially in the method I (Fig. 4a).

The difference in the average diameter of the nanofibers for various grafting methods is presented in Table 1.

**Fig. 5:**
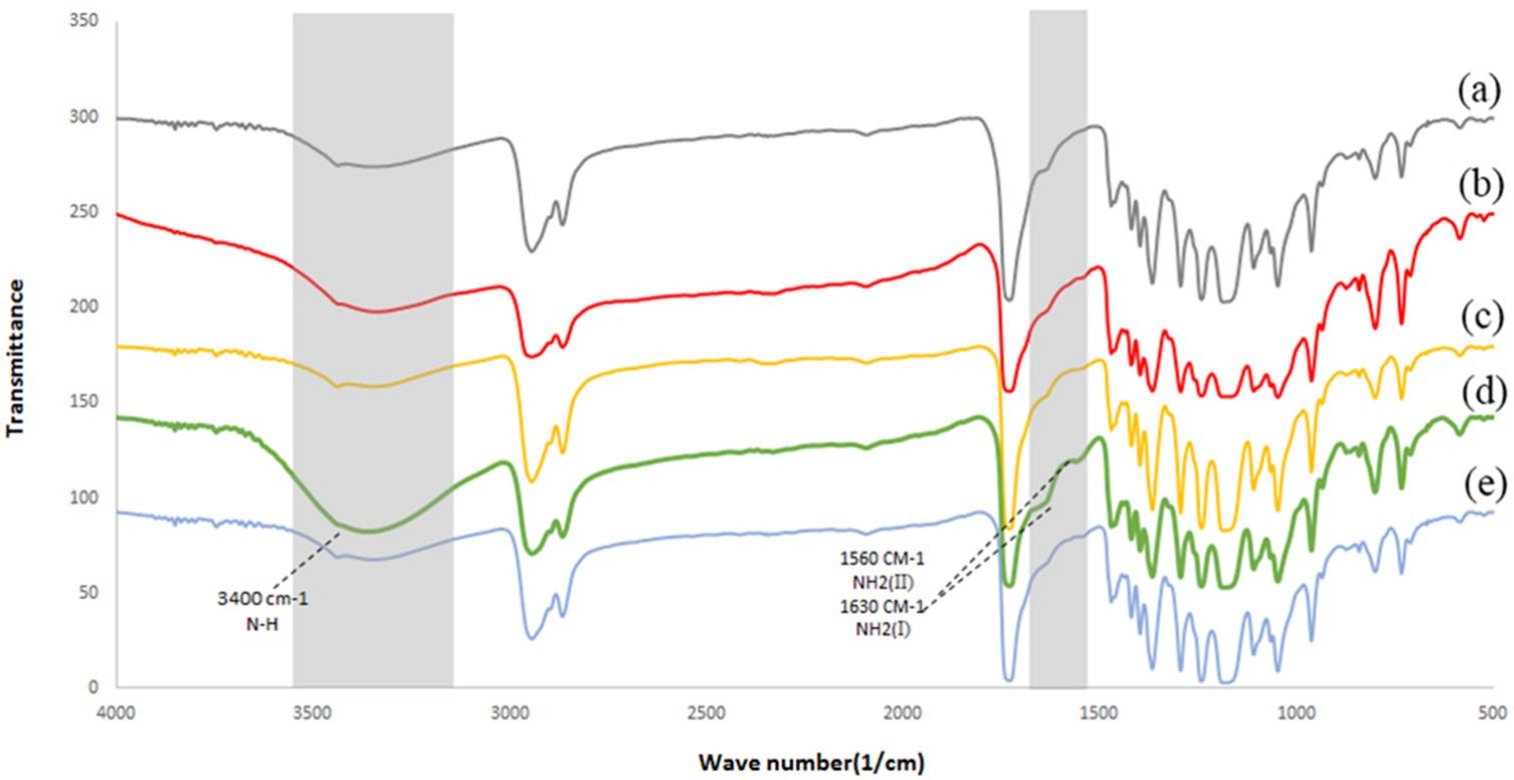
FTIR spectra of 3-min CAP-treated PCL (**a**) gelatin-grafted PCL with concentration of 3mg/mL by method I (**b**) gelatin-grafted PCL with concentration of 5 mg/m by method II (**c**) gelatin-grafted PCL with concentration of 5 mg/mL by method I (**d**) gelatin-grafted PCL with concentration of 7 mg/mL by method I (**e**)

To confirm the grafting of gelatin on the surface of the scaffold, ATR-FTIR was performed (Fig. 5). Gelatin-grafted PCL through two methods is compared to the CAP-treated sample, three distinct peaks are observed in FTIR spectra. A broader peak at 3000–3600/cm in a gelatin-grafted sample by the method I can be attributed to the stretching of the hydroxyl groups ( − OH) and NH groups from grafted gelatin macromolecules. The peak at 1630/cm shows the vibration mode of amide I groups and the last appeared peak at 1560/cm belonging to amide II (Krok-Borkowicz et al. 2015; Shen et al. 2013). Thus, the results of FTIR-ATR spectra along with the SEM images prove that the method I in grafting CAP-treated PCL scaffold is more efficient.

**Fig. 6:**
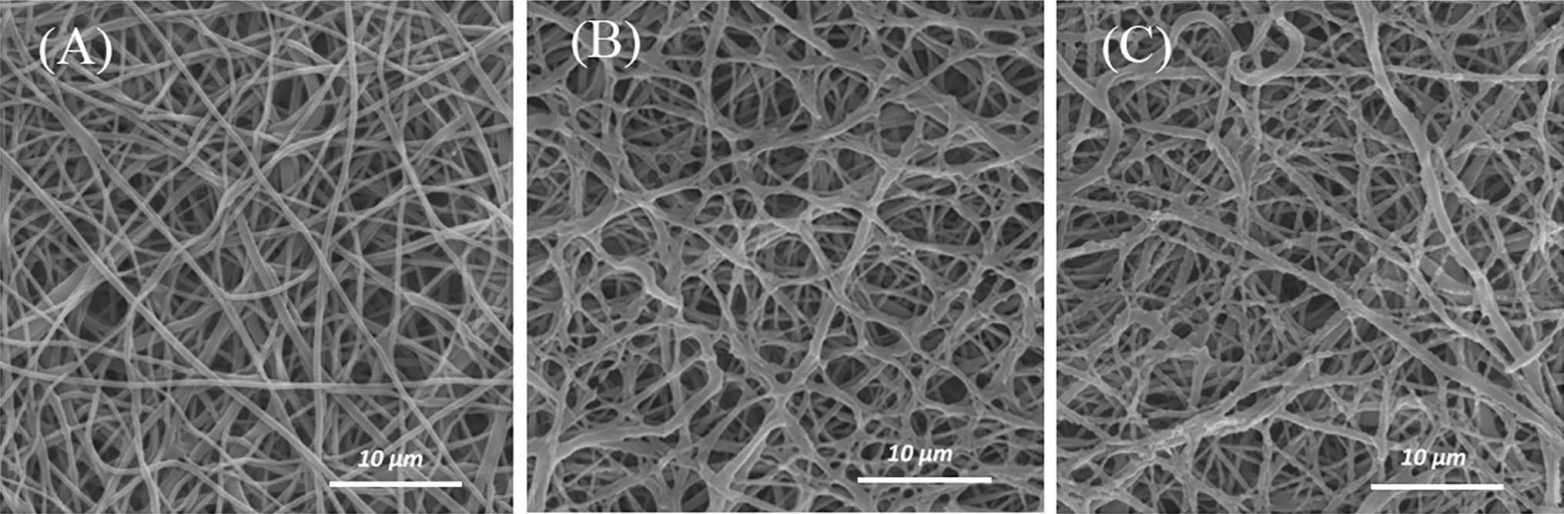
SEM images of gelatin-grafted nanofibers PCL scaffold with a concentration of 3 mg/mL (**a**), 5 mg/mL (**b**), and 7 mg/mL (**c**) by method I of grafting method on the CAP-treated scaffold (3 min)

In this study, concentrations for gelatin grafting on the surface of CAP-treated nanofibers are 3, 5, and 7 mg/mL. To examine the morphology, scaffolds grafted by the method I for different concentrations of gelatin are observed by SEM micrographs (Fig. 6a–c). Based on the results of FTIR spectra, including 3400/cm (NH groups) and 1560/cm (amide II of gelatin grafting) peaks, the concentration of 5 mg/mL is chosen as the optimal concentration for grafting the CAP-treated PCL surface (Fig. 5).

### Wettability evaluation

Suitable recognition of materials by cells needs some pre-conditions, one of which is surface wettability which leads to effective cell adhesion on the substrates. The wettability of the CAP-treated surface and gelatin-grafted PCL by CAP is studied through water contact angle test (WCA).

**Fig. 7:**
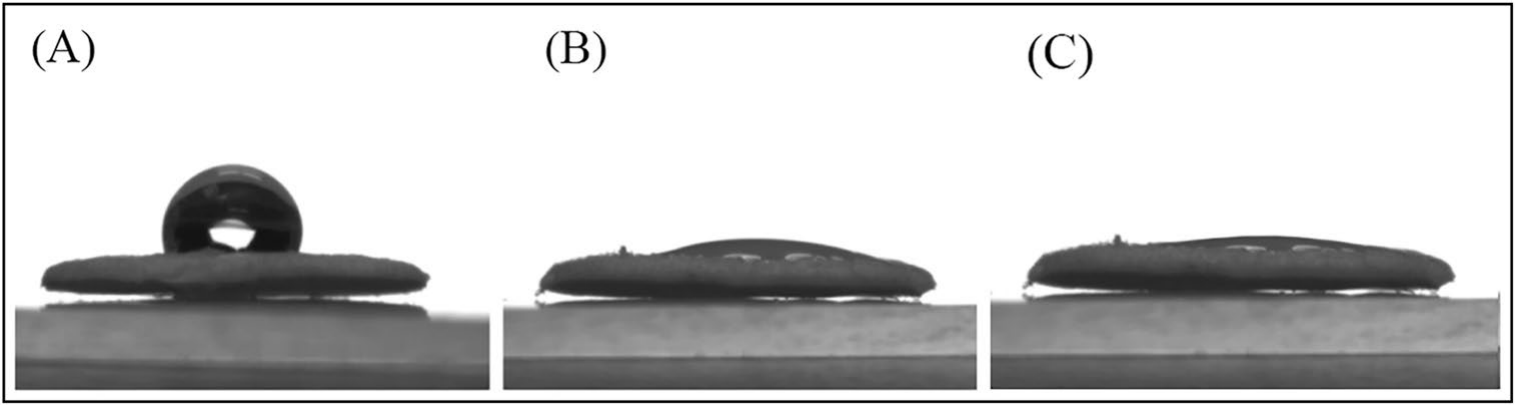
Water contact angle of PCL (**a**), CAP-treated PCL (**b**), and gelatin-grafted PCL (**c**) after 10 s

According to the water contact angles (Table 2) and the WCA images (Fig. 7), the untreated PCL nanofiber has much higher water contact angle than surface-modified PCL which means that the CAP-treated and grafted surface can effectively increase the hydrophilicity of the PCL surface. Gelatin has hydrophilic amino acids including glycine (Gly) and proline (Pro). The WCA of the gelatin grafted surface decreased by 13.7 ± 1.4 nm in comparison to the CAP-treated surface.

**Table 2:** Water contact angle (*θ*) of PCL and modified PCL; average ± SD (*n* = 5 for *θ*)

Sample	*θ*
PCL nanofibers	118 ± 4
CAP-treated PCL nanofibers	13.7 ± 1.4
Gelatin-grafted PCL nanofibers	0

### Assessment of cell adhesion and viability

**Fig. 8:**
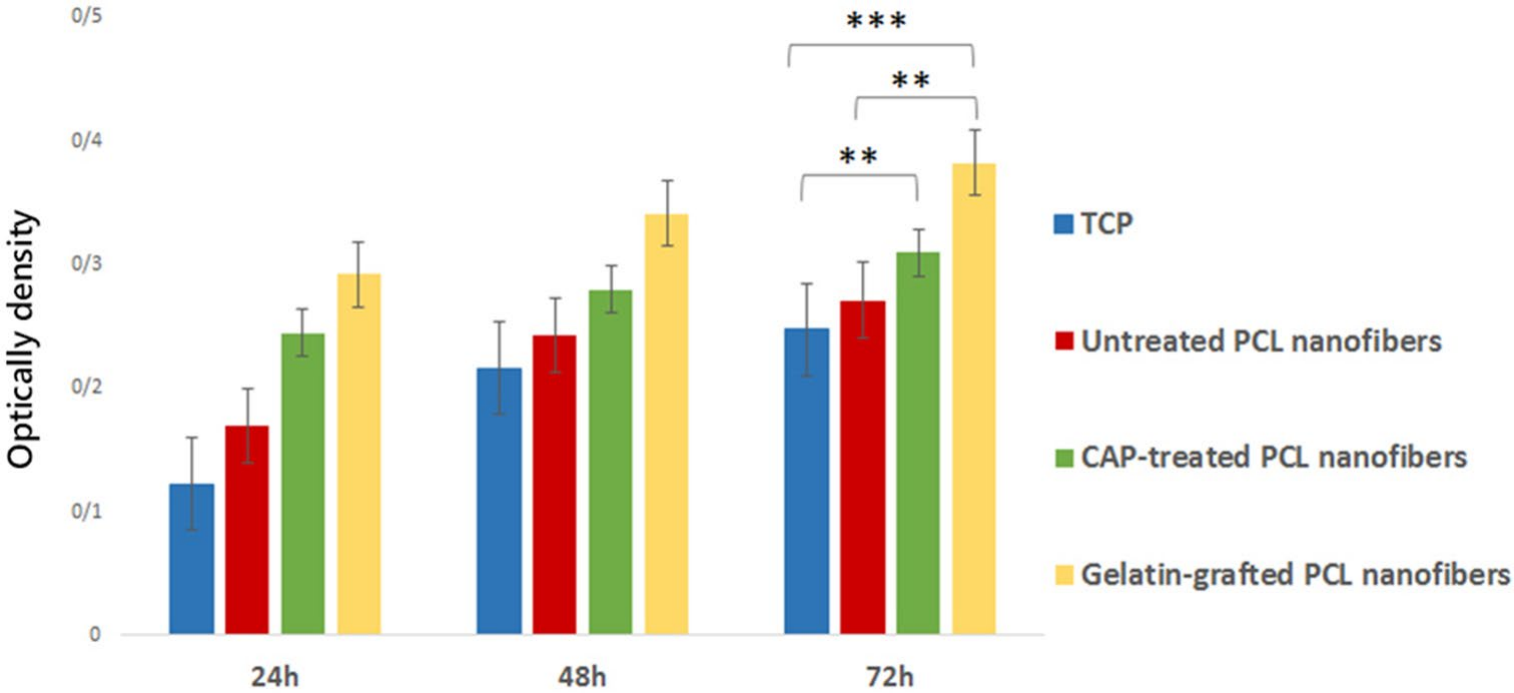
MTT assay for measuring MSCs viability on PCL, CAP-treated PCL, gelatin-grafted PCL, and TCP after 3 days of cell seeding. *P ≤ 0.05, *P ≤ 0.01, *P ≤ 0.001; mean ± SD (*n* = 3)

To evaluate the biocompatibility of the modified PCL nanofiber and its adhesion ability, MTT assay is conducted after 24, 48, and 72 h of mesenchymal stem cell culture (Fig. 8). Comparing the cell viability of cultured cells on scaffolds and TCP of MTT assay (*p* ≤ 0.05) during 3 days after MSCs cultures, a significant difference is noticeable. The results of the MTT assay shows a better rate of MSC proliferation in samples containing CAP-treated PCL and gelatin-grafted PCL than TCP and untreated PCL. The best-obtained result of MTT assay (*p* ≤ 0.05) belongs to gelatin-grafted PCL which proves the positive role of gelatin on the enhancement of cell viability.

**Fig. 9:**
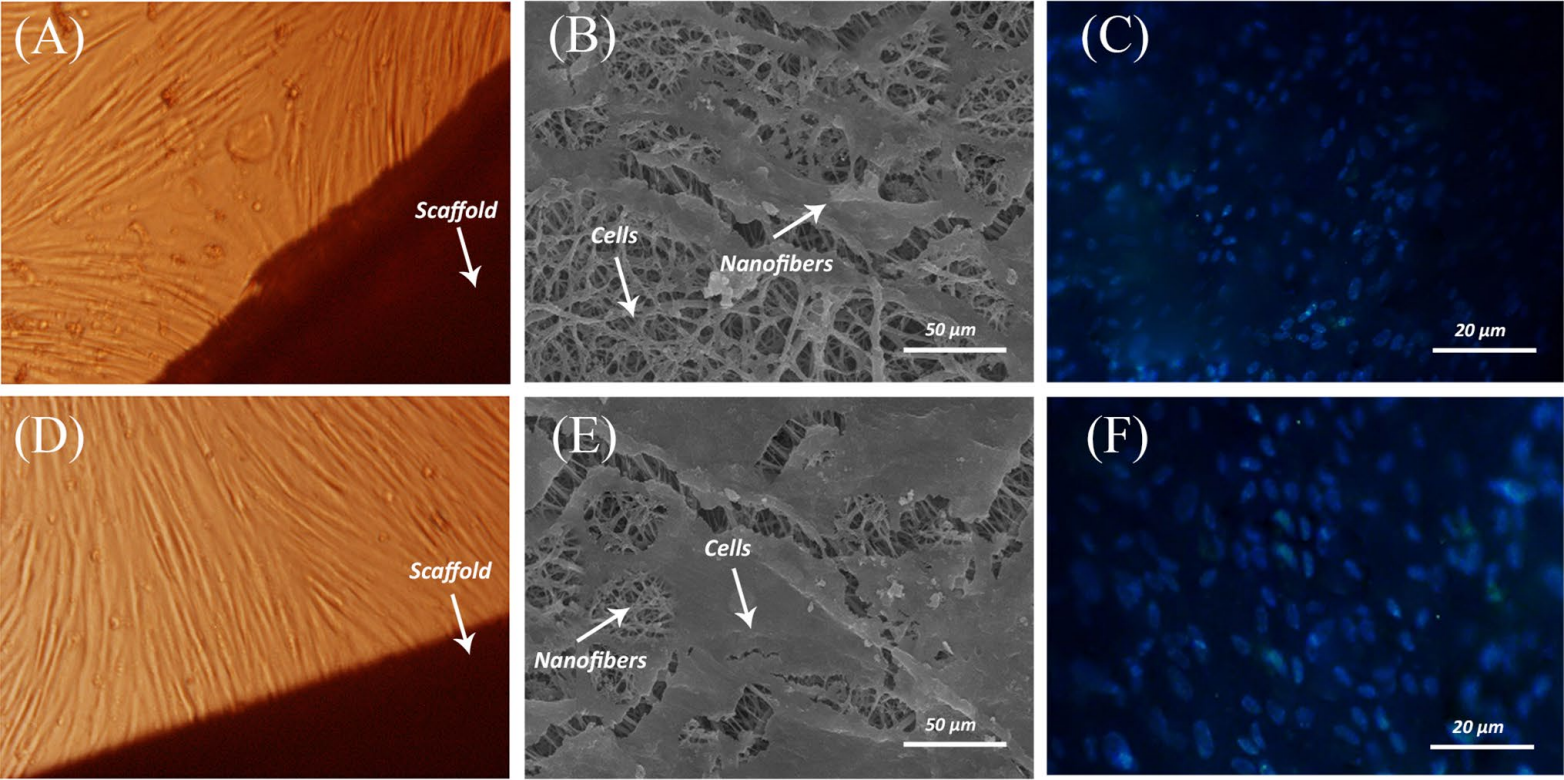
Inverted microscopy images of MSCs adjacent to the PCL (**a**) and gelatin-grafted PCL (**d**), the magnification is 200. SEM and DAPI staining after 3 days of MSCs’ culture on PCL (**b**, **c**) and gelatin-grafted PCL (**e**, **f**)

DAPI staining is performed for cultured MSCs on untreated PCL and gelatin-grafted PCL scaffold after 72 h of cell culture. The results prove a better growth and attachment of MSCs on gelatin-grafted PCL compared to untreated PCL and which is in a significant agreement with the MTT results. The images provided by the fluorescence microscope are shown in (Fig. 9c, f).

### Study of MSCs’ morphology

Invert and SEM micrographs of cultured MSCs on untreated and gelatin-grafted PCL electrospun nanofibrous after 72 h of cell culture are shown in Fig. 9. Morphology and viability of the MSCs adjacent to the untreated and gelatin-grafted PCL are observed by an inverted microscope (Fig. 9a, d). As it is shown in SEM micrographs, cells are well attached and spread on both of the scaffolds. On the other hand, according to the SEM micrographs, gelatin-grafted scaffolds have higher cell attachment and density in comparison with untreated PCL (Fig. 9b, e). As a result, gelatin-grafted nanofibrous scaffolds have better cell integration**.**

## Discussion

The previous studies have shown that one of the disadvantages of PCL scaffold is its intrinsic hydrophobic property which decreases cell adhesion on the scaffold surface and as a result, reduces the cell-scaffold interaction (Lu et al. 2014). Increasing cellular interactions with scaffolds is an important solution for tissue engineering applications. In this regard, the improvement in physical and chemical properties of the scaffold surface is one of the most important factors considered in many studies (Chong et al. 2007; Ma et al. 2007; Oyane et al. 2005; Pezeshki-Modaress et al. 2015b). In the current study, gelatin grafting using CAP treatment on the nanofiber surface is contributing factors in scaffold surface properties improvement, whose optimization leads to increased cell–scaffold interactions. Complex CAP compounds enable it to reconstruct microstructures and modify the surface properties of the scaffolds. SEM (Fig. 2) and FTIR-ATR (Fig. 3) results for CAP-treated PCL nanofibrous scaffolds reveal that 3-min treatment of the surface of the scaffold by CAP results in oxidation of the chemical groups of the surface without altering the nanofibers morphology and damaging the fibers structure. In fact, the effect of CAP treatment on changing surface properties is related to the oxidation of chemical groups existing on the scaffold surface and introduction of oxygen-containing groups such as carboxyl leading to increased hydrophilicity and surface enhancement for grafting (Li et al. 2012). The FTIR results of the gelatin-grafted PCL (5 mg/mL by the method I) in this study (Fig. 4) indicate the formation of amide groups on the surface of PCL nanofibers which expresses polymeric chains linkage of PCL with gelatin and the creation of functional groups on the surface. Gelatin through cellular connection increases the signaling pathway and cell adhesion ligands, and improves cellular attachment and proliferation. The biochemical interactions between cells and gelatin present on the nanofiber surface are through the extracellular matrix fibronectin glycoproteins. Therefore, it can be concluded that the modification of PCL nanofibers with gelatin macromolecules increases the cell–scaffold interaction by absorbing cellular cadherin molecules (Ma et al. 2005a; Schnell et al. 2007). In addition, gelatin causes PCL hydrophilization (Table 2) which can be attributed to the carboxylic and amine groups (COOH, NH) in gelatin (Li et al. 2012). Finally, the biological function of the modified surfaces is evaluated through the MTT assay (Fig. 8) and the suitable role of gelatin in the attachment and viability of the mesenchymal cells is confirmed. Gelatin-grafted PCL can provide a more suitable substrate for mesenchymal stem cell adhesion and proliferation than other groups. It is one of the most vital keys in tissue engineering applications, and techniques of biofunctionalization supporting the homogeneous cell cultures are particularly appropriate.

## Conclusion

In this study, to prepare a biofunctionalized nanofibrous substrate, the physical surface modification was performed by helium CAP treatment followed by gelatin grafting, as functional biomacromolecules, on the CAP-treated surface using glutaraldehyde as the cross-linker. FTIR-ATR results and SEM images of the modified scaffolds, respectively, showed changes in their surface chemistry with different functional groups and without changing the nanofibers morphology. WCA measurement confirmed that the wettability of the modified nanofibers was improved obviously. Therefore, the resulting scaffolds can potentially improve the cell–scaffold interactions. Mesenchymal stem cells were used to investigate the biological performance of the gelatin-grafted PCL nanofibers. It is proved by in-vitro cell adhesion and proliferation study that gelatin-grafted PCL nanofibers could provide a better environment for cell affinity and growth in comparison with the poor performance of the pristine PCL. The helium CAP treatment used in this study can be an effective method for biomacromolecule immobilization on the scaffolds in tissue engineering and providing favorable alteration of surface properties towards the optimization of cell behavior.
